# Gut-derived lactic acid enhances tryptophan to 5-hydroxytryptamine in regulation of anxiety via *Akkermansia muciniphila*

**DOI:** 10.1080/19490976.2024.2447834

**Published:** 2025-01-09

**Authors:** Miaomiao Pan, Chenglang Qian, Shaoye Huo, Yuchen Wu, Xinyi Zhao, Yueming Ying, Boyu Wang, Hao Yang, Anaguli Yeerken, Tongyao Wang, Mengwei Fu, Lihong Wang, Yuhuan Wei, Yunhua Zhao, Chunhai Shao, Huijing Wang, Chao Zhao

**Affiliations:** a MOE/NHC/CAMS Key Lab of Medical Molecular Virology, School of Basic Medical Sciences, & National Clinical Research Center for Aging and Medicine, Huashan Hospital, Shanghai Medical College, Fudan University, Shanghai, China; b Department of Clinical Nutrition, Shanghai Fifth People’s Hospital, Fudan University, Shanghai, China; c Institute of Wound Prevention and Treatment, Shanghai University of Medicine & Health Sciences, Shanghai, China; d Vanke School Pudong, Shanghai, China; e University College London, London, UK; f Department of Clinical Nutrition, Huashan Hospital, Fudan University, Shanghai, China; g Shanghai Key Laboratory of Clinical Geriatric Medicine, Huadong Hospital, Fudan University, Shanghai, China

**Keywords:** Gut microbiome, 5-hydroxytryptamine, competitive anxiety, tryptophan, lactic acid, *Akkermansia muciniphila*, propionic acid

## Abstract

The gut microbiota plays a pivotal role in anxiety regulation through pathways involving neurotransmitter production, immune signaling, and metabolic interactions. Among these, gut-derived serotonin (5-hydroxytryptamine, 5-HT), synthesized from tryptophan metabolism, has been identified as a key mediator. However, it remains unclear whether specific microbial factors regulate tryptophan metabolism to influence 5-HT production and anxiety regulation. In this study, we analyzed 110 athletes undergoing closed training and found that fecal lactate levels were significantly associated with anxiety indicators. We observed a significant negative correlation between *Akkermansia* abundance and anxiety levels in athletes. Co-supplementation with lactate and *Akkermansia muciniphila* (*A. muciniphila*) modulated tryptophan metabolism by increasing key enzyme TPH1 and reducing IDO1, thus shifting metabolism from kynurenine (Kyn) to 5-HT. In addition, lactate enhanced the propionate production capacity of *A. muciniphila*, potentially contributing to anxiety reduction in mice. Taken together, these findings suggest that enteric lactate and *A. muciniphila* collaboratively restore the imbalance in tryptophan metabolism, leading to increased 5-HT activity and alleviating anxiety phenotypes. This study highlights the intricate interplay between gut metabolites and anxiety regulation, offering potential avenues for microbiota-targeted therapeutic strategies for anxiety.

## Introduction

Anxiety disorders represent the most prevalent mental health conditions globally.^1^ Serotonin (5-hydroxytryptamine, 5-HT) plays a critical role in regulating emotions, including anxiety. It is well documented that the 5-HT system is dysfunctional in patients with anxiety disorders, with deficiencies commonly leading to negative emotions such as fear and disgust.^2^ Long-term decreases in serotonin synthesis have been linked to heightened susceptibility to social stress and mental disorders.^3–5^ In humans, serotonin is synthesized from its precursor, tryptophan.^6^ Imbalances in tryptophan metabolism can lead to not only abnormal 5-HT production but also to the accumulation of other downstream metabolites like kynurenine, contributing to psychiatric conditions.^6,7^

The primary site for the conversion of tryptophan to 5-HT is the gut, which is also the main source of peripheral serotonin.^6^ Research has demonstrated that tryptophan metabolism is intricately regulated by the symbiotic gut microbiota. In a germ-free environment, mice exhibit elevated serum tryptophan levels.^8^ This suggests that microbes play a crucial role in maintaining the metabolic balance of tryptophan, but whether this process is involved in the occurrence of anxiety is unknown. Emerging evidence highlights the crucial role of the gut microbiome and its metabolites in modulating anxiety.^9,10^ The composition of the gut microbiota is heavily influenced by factors such as diet, physiological regulation, and the external environment, making studies on the physiological functions of microorganisms somewhat limited.

Elite athletes experience unique and intense stressors such as risks of injury and public scrutiny.^11–13^ The highly controlled diets, training routines, and rest patterns of athletes minimize the influence of confounding physiological factors, making them an ideal cohort for investigating the specific mechanisms underlying anxiety. This study focuses on Chinese shooting athletes in closed training environments, allowing for an exploration of their distinctive gut microbiota. Our findings indicated that athletes with lower anxiety levels exhibited increased fecal lactate levels. We further demonstrated that lactate increases the levels of *Akkermansia*, which is negatively correlated with anxiety, and together they improve the imbalance in tryptophan metabolism. We found that *A. muciniphila* increases propionic acid levels in a lactate-enriched gut environment, which is sufficient to alleviate anxiety phenotypes and restore gut tryptophan metabolism imbalance. In summary, our findings indicate that enteric lactate, in conjunction with *A. muciniphila*, collaboratively ameliorates anxiety-related imbalances in tryptophan metabolism.

## Material and methods

### Participants and procedures

Participants, aged between 14 and 34 years (*n* = 110), were systematically enlisted from the Shanghai Shooting and Archery Sports Center, adhering to stringent inclusion criteria: absence of probiotic preparation used at study onset; no engagement in clinical trials within the preceding 6 months; no antibiotics or non-steroidal anti-inflammatory drugs in the past 3 months; no preexisting conditions such as diabetes, autoimmune, or gastrointestinal disorders like inflammatory bowel disease. All athletes underwent 1–2 rounds of interviews where detailed clinical information was collected. Generalized Anxiety Disorder Scale (GAD-7), State-Anxiety Inventory – trait Version (STAI-T) and Competitive State Anxiety Inventory-2 (CSAI-2) were used to assess the athletes’ anxiety profiles.^14–16^ Thresholds for mild and moderate-to-severe anxiety were established with GAD-7 scores exceeding 4 and 9, respectively.^17^ Questionnaires for all participants were completed with one-to-one guidance from a physician. Self-assessment, clinician assessment of detailed diet and trainer statistics of exercise performance were also conducted. Fecal specimens were collected during the morning at the Shanghai Shooting and Archery Sports Center, promptly secured in aseptic fecal collection tubes, subjected to flash-freezing in liquid nitrogen, and stored at −80°C within 3 h post-collection. Ethical clearance for this study was granted by the Ethics Committee of Shanghai Fifth People’s Hospital, Fudan University, with all participants providing written informed consent (Approval No. 2020002X1).

### Copyright permission statement

The authors acknowledge that the State-Anxiety Inventory – trait Version used in this study is the property of the mind garden (www.mindgarden.com). Permission to use and reproduce this questionnaire was granted by the mind garden. The authors are grateful for the company’s cooperation in allowing the inclusion of this tool in the research.

### Animal studies

#### General

Six-week-old male C57BL/6N mice were purchased from Charles River (Beijing, China) and adaptively housed for 1 week before the experiment. Animals were housed in a temperature- and humidity-controlled room (23–25°C, 50–60%) under a 12:12 h light–dark cycle with free access to food and water. Animals were randomized into experimental groups. The protocols for animals were approved by the animal ethics committee of the School of Basic Medical Sciences, Fudan University (No. 20220627–001).

#### Aerobic exercise training procedure

The mice were randomly assigned into the Control (Ctrl) group, Lactate (Lact) group, and Exercise (EX) group. Mice in the Lact group received daily intraperitoneal injections of sodium lactate (750 mg/kg, MCE, New Jersey, USA). Control and EX group were injected with 0.9% saline solution vehicles. The mice underwent a two-week aerobic conditioning regimen on a motorized treadmill (RWD80805A, RWD, Shenzhen, China), under the incentive of an electrified grid. Acclimatization to the exercise regimen began with a 10 m/min pace for 2 days, followed by an escalating protocol commencing at 10 m/min for 30 min on the first day and 45 min on the subsequent day. The training frequency was 5 days per week interspersed with 2 days of rest, spanning a complete two-week cycle. The regimen was adapted from prior protocols to align with moderate exercise intensity, corresponding to 65–70% of the maximal oxygen consumption rate.^18^

#### Rotarod test

Motor coordination and balance were assessed using an accelerated rotating rod device (VanBi, FanBi, Shanghai, China). The device consisted of a rotating rod, 3 cm in diameter, that was divided into six running lanes, allowing up to six mice to be tested simultaneously. The day before the test, the animals were placed on a cylinder rotating at a fixed speed of 4 revolutions per minute (r.p.m.) for two consecutive 90-s intervals separated by a 2-h break to acclimatize to the rotating bar. On the day of the test, the animals were placed on the rotating cylinder, and the time each animal was able to maintain balance was recorded. The rotating pole was set to accelerate from 0 to 40 rpm in a linear fashion over a 5-min period. Falling latency was measured in seconds after the mouse either fell off the pole, remained attached to the pole for two consecutive rotations, or rotated three times in 10 s. The maximum latency was 300 s when the mouse did not fall from the pole or make any rotational errors described above.^19^

#### Chronic unforeseeable mild stress stimulation (CUMS) procedure

The mice were randomly assigned into the Control (Ctrl) group, the chronic unforeseeable mild stress stimulation (CUMS) group, Lactate-treated CUMS (CUMS-lact) group, and Lactate (Lact) group. All mice were given a four-week CUMS paradigm, except for the Ctrl and Lact groups.^20^ Mice in the CUMS-lact and Lact group were intraperitoneally injected with sodium lactate daily (750 mg/kg, MCE, New Jersey, USA) during the stress intervention.^21^ Control and CUMS groups were injected with 0.9% saline solution vehicles. Fresh fecal samples were collected on day 28. After the completion of the behavioral test, all tissues were collected within approximately 2 h and stored at −80°C.

#### ABX treatment and bacterial colonization

The mice were administered with an antibiotic cocktail containing ampicillin, neomycin, metronidazole, and vancomycin via drinking water for 7 days in antibiotic treatment (ABX) groups. *Akkermansia muciniphila* (*A. muciniphila*, ATCC BAA-835) was anaerobically cultured in brain heart infusion (BHI) broth supplemented with 0.5% gastric mucin (MCE, New Jersey, USA) at 37°C. Mice were randomly divided into three groups for *A. muciniphila* administration: ABX+Akk, ABX+Akk+Lact, and ABX+Heat-killed Akk. Prior to *A. muciniphila* transplantation, all mice underwent a 7-day ABX treatment, followed by daily intragastric administration of 2 × 10^8 cfu/200 μL live *A. muciniphila*, an equivalent volume and CFU count of heat-killed *A. muciniphila* (70°C for 15 min), or an equal volume of sterile PBS containing 25% glycerol (1×). Mice in groups requiring lactate treatment (i.e., ABX+Lact and ABX+Akk+Lact) received treatment according to previous protocol. For the oral propionate administration group (ABX+calcium propionate), a 7-day regimen of calcium propionate (200 mM, MCE, New Jersey, USA) was applied.

Fecal samples were collected before, and at 48 h and 7 days post-colonization in all Akk groups. The absolute quantitative PCR (qPCR) was performed using primers specific for *A. muciniphila*: forward CCTTGCGGTTGGCTTCAGAT, reverse CAGCACGTGAAGGTGGGGAC. A series of diluted target genes of *A. muciniphila* were used to generate standard curve. The copy number of *A. muciniphila* in each sample was normalized to fecal weight. Total 16S rRNA was also quantified and normalized using universal bacterial primers: forward ACTCCTACGGGAGGCAGCAG, reverse ATTACCGCGGCTGCTGG.^22,23^

#### Germ-free mice

Six-week-old male C57BL/6J mice were purchased from Gnotobio (Guangdong, China) and were maintained in sterile isolators under gnotobiotic conditions. Sterilized, nutrient-complete rodent chow and autoclaved or irradiated water were provided to the mice. Temperature, humidity, and light cycles within the isolators were maintained at standard laboratory levels, with temperature set at 20–22°C and a 12-h light–dark cycle. The *A. muciniphila* solution is prepared as described above and administered to the mice by gavage inside the isolator.

#### Behavioral tests

The mice were placed in the laboratory for 2 h before all behavioral tests. All mice were tested in the same order.

Open-field test (OFT): mice were placed individually in the corner of an open-field box (length × width × height, 50 cm × 50 cm × 50 cm, VanBi-OF, FanBi, Shanghai, China). The mice were allowed to explore freely for 6 min, and the spontaneous activities over the last 5 min were recorded. After each animal was tested, the instrument was cleaned with ethanol to remove olfactory cues.^24^

Elevated plus maze test (EPMT): The device was 100 cm above ground, including two closed arms with dark walls (L × W × H, 60 cm × 10 cm × 40 cm) and two open arms (L × W, 60 cm × 10 cm, VanBi, FanBi, Shanghai, China). The four arms were connected by the central platform (L × W, 10 cm × 10 cm). Mice were placed in the center area and then allowed to explore the maze for 5 min, defined as entering an arm when all four paws crossed the dividing line. The time and entries into the arms were measured as indicators of anxiety. After each test, the maze was cleaned with ethanol to remove olfactory cues.^25^

### UHPLC-MS/MS analysis

UHPLC-MS/MS analyses were performed using a Thermo Syncronis C18 (2.1 mm × 100 mm, 1.7 μm) UHPLC system (ThermoFisher, Germany) coupled with an Orbitrap Q Exactive^TM^ series mass spectrometer (Thermo Fisher, Germany) in Scale Co., Ltd. (Beijing, China). Samples were injected onto a Hypesil Gold column (2.1 mm × 100 mm, 1.7 μm) using an 18-min linear gradient at a flow rate of 0.2 mL/min. The eluents A and B were 0.1% FA in water and acetonitrile, respectively. The solvent gradient was set as follows: 0 ~ 1 min, 95% A; 1 ~ 5 min, 95%~40% A; 5 ~ 8 min, 40%~0% A; 8 ~ 11 min, 0% A; 11 ~ 14 min, 0%~40% A; 11 ~ 15 min, 40%~95% A; 15 ~ 18 min, 95% A. Q Exactive^TM^ series mass spectrometer was operated in positive/negative polarity mode with spray voltage of 3.2 kV, capillary temperature of 320°C, sheath gas flow rate of 40 arb, and aux gas flow rate of 10 arb.

### qPCR analysis

Total RNA was extracted from mouse colon pieces using TRIZOL (Invitrogen, California, USA). Chloroform and isopropanol were utilized to precipitate the aqueous phase, and RNA concentration was determined using NanoDrop 2000 (Thermo Fisher, Massachusetts, USA). cDNAs were prepared from 800 ng of total RNA using reverse transcription kit (Promega, Shanghai, China), followed by qPCR analysis employing SYBR green kit (Promega, Shanghai, China).

### Western blotting

Clean colon tissues (2–3 cm) were collected in 2 mL screw-capped microtubes containing two steel beads and 200 μL SDS lysis buffer (50 mm tris (pH 8.1), 1 mm EDTA, 1% SDS, 1 mm fresh dithiothreitol, sodium fluoride, leupeptin) supplemented with protease inhibitor cocktail (Sigma-Aldrich, Wisconsin, USA). Homogenization (4800 rpm, 10 s, 6 cycles) was performed, and then the lysed mixture was centrifuged at 12,000 rpm for 15 min at 4°C. The supernatant was transferred into 1.5 mL Eppendorf (Ep) tubes, and protein concentration was determined using the Bicinchoninic Acid (BCA) Protein Assay Kit (Solarbio, Beijing, China). Tissue lysates boiled with 6 × loading buffer (Beyotime, Shanghai, China) were loaded onto 10% or 12.5% SDS-polyacrylamide gradient gel (Genscript, Nanjing, China) and transferred onto PVDF membranes (Millipore, Massachusetts, USA) through a liquid transfer system (Genscript, Nanjing, China). The membranes were then probed with diluted primary antibodies overnight at 4°C, after blocking with 3% bovine serum albumin (BSA) in tris-buffered saline and Tween 20 (TBST; 10 mm Tris-base pH 7.6, 150 mm NaCl, and 0.1% (v/v) Tween 20) at RT for 2 h. After washing with TBST, the bands were incubated with secondary antibodies in blocking buffer (1:10,000) for 2 h at RT. Visualization was then performed using a chemiluminescent HRP substrate (Millipore, USA). The antibodies used in western blotting were listed as follows: anti-IDO1 (Cell Signaling Technology Cat#51851S, AB_2799402), anti-KMO (Proteintech Cat#10698–1-AP, AB_2296744, Wuhan, China), anti-3HAO (Proteintech Cat#12791–1-AP, AB_2115432, Wuhan, China), anti-MCT1 (Proteintech Cat#20139–1-AP, AB_2878645, Wuhan, China), anti-GAPDH (Proteintech Cat#HRP-60004, 2737588, Wuhan, China), anti-TPH (Abcam Cat# ab52954, AB_2207555, Cambridge, UK), anti-TPH1 (HUABIO Cat#ET1610-37, AB_3069921, Hangzhou, China), anti-claudin-1 (Abcam Cat#ab307692, AB_3083082, Cambridge, UK).

### ELISA immunoassays

The metabolite assays from the prefrontal cortex and serum were carried out according to the instructions of the manufacturer. The ELISA kits included 5-HT (E-EL-0033c, Elabscience, Wuhan, China) and lactate (E-BC-Ko44-M, Elabscience, Wuhan, China). Data were normalized based on protein concentrations determined by BCA.

### HE staining for histology

Four percent paraformaldehyde-fixed intestinal samples were embedded in paraffin, sectioned at 5 μm thickness and stained with hematoxylin and eosin (H&E). Photomicrographs were scanned and observed via ZEN3.1 (Zeiss, Germany).

### Immunofluorescence

The distal colon tissues were tested in this assay. Perfused tissues were immersed in 4% paraformaldehyde at 4°C overnight, then embedded in paraffin and cut into 3–4-μm-thick sections. After dewaxing and antigen retrieval, sections were blocked with 5% BSA buffer for 2 h at RT, followed by incubation with primary antibodies diluted in blocking solution (IDO1 1:100, 51851S, Cell Signaling Technology; 5-HT 1:500, ab66047, Abcam; CgA 1:200, ab45179, Abcam; Claudin-1 1:500, GB14066–50, Servicebio) at 4°C overnight. After three washes, the sections were further incubated with secondary antibodies for 2 h at RT, shielded from light. DAPI solution was then applied before the sections were sealed with glycerol. Samples were imaged by confocal microscopy using Pannoramic MIDI (3D HISTECH, Hungary) and images were exported and analyzed using software CaseViewer2.4 (https://www.3dhistech.com/solutions/caseviewer/) and ImageJ (https://imagej.nih.gov/ij/), respectively.

### Biological sample collection and analysis

The 16S rRNA V3-V4 region was amplified by PCR and then sequenced using lon S5 XL/Illumina platform (Thermo Fisher, Massachusetts, USA). Sequences were denoised by unoise3 to obtain single-base precision amplicon sequence variants (ASVs).^26^ We then used vsearch plugin to cluster sequences at 97% identity and the taxonomy was assigned against the SILVA database (V.123, https://www.arb-silva.de/). The primary analyses for microbiome differences included Alpha-diversity (Simpson, Shannon, ACE, and Chao1 indices) and Beta-diversity (Bray-Curtis dissimilarity). Principal coordinate analysis (PCoA) plots were created for visualization of the Beta-diversity distance between each sample. Linear discriminant analysis effect size (LEfSe) analysis was carried out to identify differentially abundant taxa between groups. In this method, features with significant distinct abundance were first detected using a non-parametric factorial Kruskal-Wallis and sum-rank test, then a linear discriminant analysis (LDA) was performed to calculate the effect size of each feature. We used Phylogenetic Investigation of Communities by Reconstruction of Unobserved States2 (PICRUSt2) to populate the metagenomes of the gut microbiome from 16S rRNA sequences. LEfSe and PICRUSt2 module were both from the Galaxy (http://galaxy.biobakery.org). Statistical differences in functional items were assessed by one-way ANOVA using statistical analysis of Metagenomic Profiles software (STAMP v2.1.3, Nova Scotia, Canada).

For transcriptome sequencing of RNA, total RNA was enriched by Oligo(dT) magnetic beads using the NEBNext Ultra II RNA Library Prep Kit. cDNA was synthesized by random interruption of fragmented mRNAs as templates, and random oligonucleotides as primers, and the libraries were amplified by PCR. Multiplexed DNA libraries are homogenized and mixed in equal volumes. The mixed libraries were gradually diluted and quantified, and then sequenced in PE150 mode on an Illumina sequencer. The downstream data were filtered and sequenced to generate annotation files, and finally the expression was normalized by HTSeq (v0.9.1) and FPKM.

### Statistical analysis

Statistical analyses were performed with R (V.4.4). Continuous variables were expressed as mean ± standard deviation (SD) of the mean or median, and the categorical variables were described as proportions. Differences in alpha diversity were verified by Wilcoxon analysis. The relationship between community composition and exogenous elements of the microbiome was analyzed using PERMANOVA and VEGAN R packages. *p* values were adjusted with the use of the Benjamini–Hochberg procedure to correct for multiple comparisons, with a false discovery rate (FDR) of 0.05. The Shapiro-Wilk and Levene tests were used to test the assumptions of normality and homogeneity of variance, respectively. Other statistical analyses included the Mann–Whitney U-test, Kruskal–Wallis test, Student’s t-test, Pearson’s correlation, and Spearman’s rank correlation (GraphPad Prism9, La Jolla, CA, USA). All the tests were 2-tailed and *p* < 0.05 was designated as a significant statistical difference.The Figs. 2a, 2d, 3a, 5a and 7 in this paper were created with BioRender (https://BioRender.com).

## Results

### Gut microbiome composition and lactate is associated with anxiety in athletes

We conducted an examination of 110 professional shooters undergoing closed training. Their diet, training regimen, and schedules were strictly controlled, providing an ideal cohort for studying gut microbiota. The basic characteristics of these participants are outlined in Table S1. Anxiety levels were assessed using the GAD-7 scoring system. Athletes with generalized anxiety exhibit higher levels of trait anxiety and more pronounced pre-competition anxiety (Figure S1a). To further investigate the microbial characteristics associated with anxiety, we analyzed the fecal samples of athletes.

**Figure 1.**
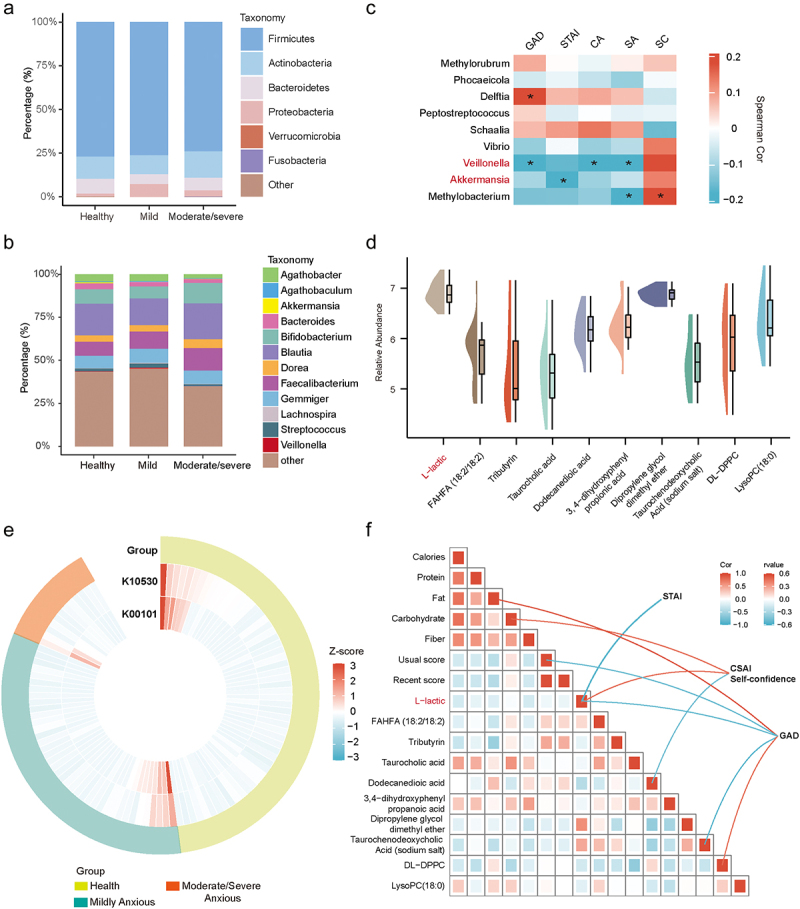
Anxiety associated with gut microbiome composition and fecal lactate.

Utilizing 16S rRNA sequencing, we identified 204 ASVs across three groups (health, mildly and moderate-to-severe anxious). We found that the relative abundance of *Firmicutes* gradually decreases with increasing levels of anxiety (Figures 1a and S1b,c). In individuals with moderate-to-severe anxiety, the proportions of *Agathobacter*, *Akkermansia*, and *Bacteroides* declined, whereas *Faecalibacterium* and *Dorea* were more prevalent (Figure 1b). Linear discriminant analysis effect size (LEfSe) identified distinct microbial markers between groups (*p* < 0.05, linear discriminant analysis [LDA] scores > 2) (Figure S1d). In total, we identified 33 discriminant taxa, primarily classified into 25 genera (Figure S1e). To identify the microbes most closely linked to anxiety indicators, we conducted a correlation analysis. The results showed that *Delftia*, *Veillonella*, *Akkermansia*, and *Methylobacterium* exhibited significant correlations with anxiety (Figure 1c). Notably, *Veillonella* and *Akkermansia* have recently been shown to play important roles in lactate metabolism cycling in the human gut.^21,27^ We also identified lactate among the differential metabolites in fecal samples (Figure 1d). The results showed a decrease in L-lactic in the moderate/severe anxiety group (Figure S1f). Additionally, we screened for gene functional clusters potentially involved in lactate cycling based on PICRUSt2 and identified two KO genes: K10530 (L-lactate dehydrogenase cytochrome [EC:1.1.2.3]) and K00101 (L-lactate oxidase [EC:1.1.3.2]). As anxiety levels increase, these two KO genes show a downward trend, suggesting alterations in gut lactate metabolism activity (Figure 1e). Finally, we conducted a correlation analysis between various factors, including diet and performance, and anxiety levels in athletes. Among these, fecal lactate levels emerged as a key factor associated with anxiety levels (Figure 1f).

(a,b) Comparative analysis of gut microbial composition at the phylum (a) and genus (b) levels across the three groups. (c) Heatmap depicting correlations between differential microbial taxa and anxiety scale scores (*,*p* < 0.05). GAD: Generalized Anxiety Disorder; STAI: State-trait anxiety inventory; CA: Cognitive Anxiety; SA: Somatic Anxiety; SC: Self-Confidence. (d) Top 10 differential metabolites identified in non-targeted metabolomic analysis of athlete feces. (e) Annular heatmap illustrating the relative abundance of KO genes K00101 and K10530 associated with lactate metabolism, based on PICRUSt2. (f) The correlation interaction network heatmap illustrates the relationships between athletes’ psychological scale scores, dietary components, athletic performance, and differential fecal metabolites (*p* < 0.05). Blue lines represent negative correlations, while red lines indicate positive correlations.

### Lactate enhanced the intestinal 5-HT system in mice

**Figure 2.**
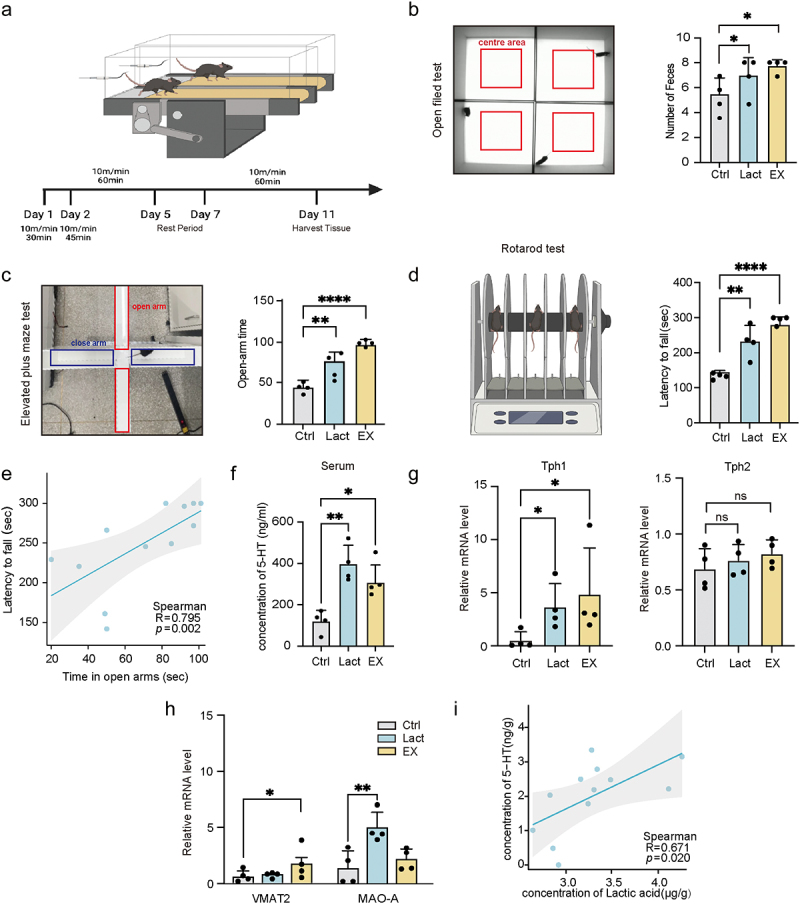
Lactate enhanced the activity of 5-HT system in mice.

Next, we focused on the effect of local intestinal lactate on anxiety. Given that systemic lactate can be rapidly absorbed by the intestines, we opted to modify local lactate levels in the gut through intraperitoneal injection.^21^ In addition, regular exercise significantly alleviated anxiety in mice, with exercising mice serving as a positive control in this study (Figure 2a).^21,28,29^ Similar to the effects of exercising, lactate treatment resulted in reduced anxiety-related behaviors in both the open field test and the Elevated Plus Maze Test (EPMT) (Figure 2b,c). Interestingly, lactate also improved fatigue tolerance and balance in mice (Figure 2d). This improvement was strongly correlated with reduced anxiety levels (*p* = 0.002, *R* = 0.795) (Figure 2e).

(a) Schematic representation of the experimental design for the mouse locomotion model. (b,c) Illustrations of the open field (b) and elevated plus maze tests (c), with accompanying bar charts showing statistical analysis of defecation frequency and time spent in the open arms. (d) Comparative analysis of fall times in the rotating rod system across the three groups (*n* = 4). (e) Spearman’s rank correlation between drop time and time spent in the open arms. (f) Measurement of serotonin (5-HT) levels in serum. (g) Gene expression profiles of tryptophan hydroxylase 1 (*Tph1*) in the colon and tryptophan hydroxylase 2 (*Tph2*) in the prefrontal cortex of mice, normalized to 18S rRNA. (h) Gene expression profiles of serotonin metabolism pathways in the mouse colon, normalized to 18S rRNA, including vesicular monoamine transporter 2 (VMAT2) and monoamine oxidase A (MAO-A). (i) Spearman’s rank correlation between 5-HT and lactate concentrations in the mouse colon. Data are presented as mean ± SD, with significance indicated by *p < 0.05; **p < 0.01; ***p < 0.001; ****p < 0.0001; ns, no significance.

Considering that anxiety is associated with imbalances in serotonin (5-HT) production, we compared peripheral 5-HT levels across the three groups. The results showed that both physical exercise and lactate administration significantly increased serum 5-HT levels (Figure 2f). Serotonin synthesis is regulated by two rate-limiting isozymes: tryptophan hydroxylase 1 (TPH1), primarily expressed in intestinal enterochromaffin (EC) cells, and tryptophan hydroxylase 2 (TPH2) mainly found in the central nervous system.^30,31^ We compared the levels of *Tph1* in the gut and *Tph2* in the prefrontal cortex. The lactate-induced changes in this enzyme expression occurred only in the gut (Figure 2g). Furthermore, the other two enzymes involved in serotonin regulation – monoamine oxidase A (MAO-A) and vesicular monoamine transporter 2 (VMAT2), exhibited significant changes in the colon of treated mice, but not in the brain (Figures 2h and S2a). The concentration of 5-HT in colon tissues increased, in response to elevated intestinal lactate levels, supporting the hypothesis that local intestinal lactate affects the activity of the gut serotonin system (Figure 2i). These findings suggest that lactate exerts significant anxiolytic effects and influences both local enteric and systemic serotonin levels by enhancing serotonin synthesis in the colon.

### Lactate alleviated chronic anxiety and reshaped the microbiome

**Figure 3.**
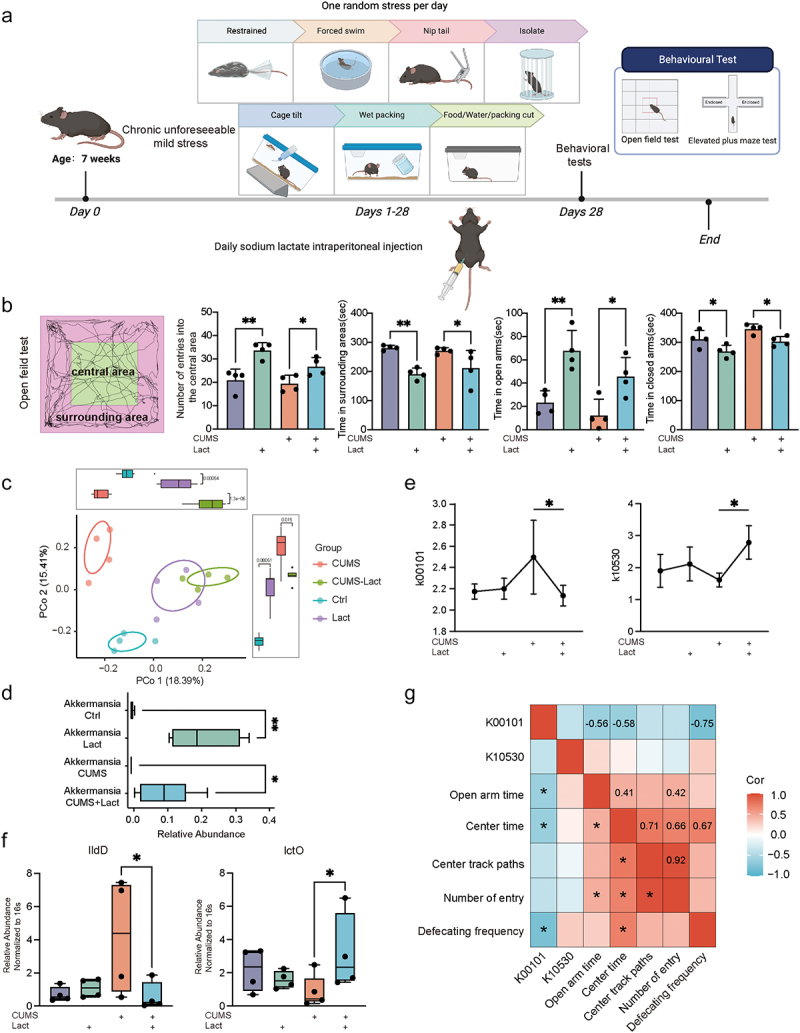
Lactate alleviated anxiety and modulated gut microbiota in mice.

To further investigate the effects of lactate on chronic anxiety processes, we established a chronic unforeseeable mild stress (CUMS) model in mice (Figure 3a). Lactate increased exploratory behavior in mice and reduced their avoidance behavior in elevated and exposed areas (Figure 3b). Based on previously observed anxiety-related microbial changes in athletes, we further investigated whether lactate could simultaneously affect the gut microbiome of anxious mice. Principal Coordinates Analysis (PCoA) revealed distinct microbial community structure, indicating that intestinal lactate directly impacted the gut microbiota (Figure 3c). Lactate restored the abundance of certain microorganisms that were significantly altered by chronic stress, including *Lactobacillus*, *Alistipes*, *Ruminiclostridium*, and *Prevotellaceae* (Figure S2b). Notably, the relative abundance of *Akkermansia* increased significantly under lactate treatment (Figure 3d). However, another anxiety-related genus observed in athletes, *Veillonella,* showed no significant change after lactate treatment (Figure S2c).

(a) Experimental design and behavioral recording protocol for the mouse chronic unforeseeable mild stress model. (b) Outcomes of the open field test and elevated plus maze test, highlighting time spent in the surrounding field, open arms, closed arms, and frequency of entries into the central area. (c) Principal Coordinate Analysis (PCoA) of gut microbiota beta diversity in mice, based on Bray-Curtis dissimilarity. (d) Relative abundance of *Akkermansia* in mouse gut microbiota from different groups. (e) Relative abundance of KO genes K00101 and K10530 related to lactate metabolism in different groups, data were obtained by PICRUSt2. (f) Relative abundance of *lldD* and *lctO*, representative genes of K00101 and K10530, respectively, were verified by qPCR of fecal DNA. 16s rDNA were used for exogenous control. (g) Correlation coefficients between behavioral indicators of anxiety in mice and specific function gene, with positive correlations shown in red and negative correlations in blue. The filling ratio in the boxes represents the strength of the correlation coefficient between the variables. Data are presented as mean ± SD. *p < 0.05; **p < 0.01; ***p < 0.001.

Based on our previous result that anxiety may involve the activity of the intestinal lactate cycle, we compared changes in functional gene clusters in the gut of mice with and without lactate treatment. Lactate significantly altered the abundance of KO genes K00101 and K10530 in the CUMS model (Figure 3e). This effect was further validated by quantifying the abundance of their representative genes of K00101 and K10530, *lldD* and *lctO*, respectively, using qPCR of fecal DNA samples (Figure 3f).^32^ Importantly, lactate reduced the abundance of K00101, which exhibited a significant negative correlation with the reduction in anxiety levels in mice (Figure 3g).

### Lactate enhanced the metabolic shift of tryptophan metabolism towards 5-HT synthesis

**Figure 4.**
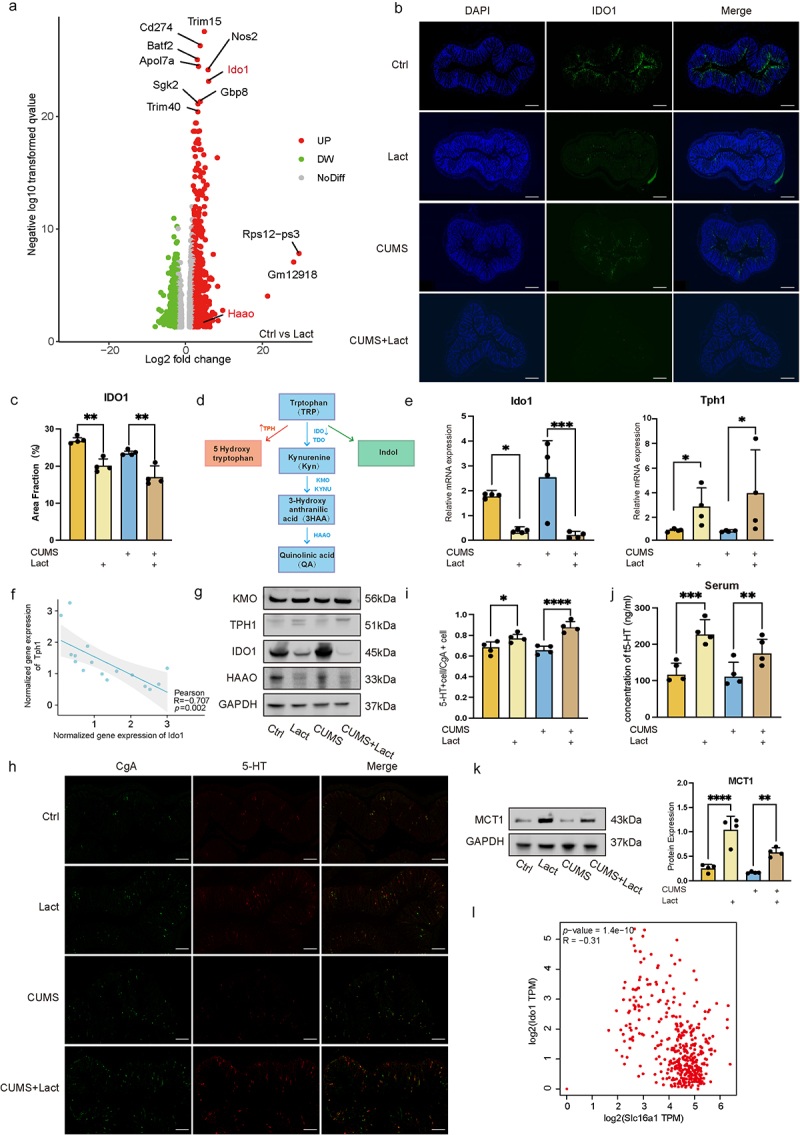
Lactate redirected tryptophan metabolism through enzymatic regulation.

Building on previous findings that lactate primarily boosts serotonin (5-HT) production in the gut, we delved deeper into the mechanisms by which lactate ameliorates chronic anxiety by performing RNA sequencing analysis of the mouse colon. Intergroup comparisons showed significant downregulation of Indoleamine 2,3-dioxygenase 1 (*Ido1*) in response to lactate (Figures 4a, S3a). IDO1, typically highly expressed in intestinal tissues,^33^ was inhibited by lactate (Figure 4b,c). As a key enzyme, IDO1 dictates the metabolism of 95% of the free tryptophan through the kynurenine (Kyn) pathway.^34^ Alterations in tryptophan metabolism can affect the production of neurotransmitters downstream.^35^ Our results confirmed that lactate alters circulating 5-HT levels (Figure 2f). Therefore, we hypothesize that lactate redirects tryptophan metabolism, favoring the conversion from Kyn to the 5-HT pathway (Figure 4d). Comparative analyses of the expression of IDO1 and TPH1 indicated opposite trends (Figures 4e-g, S3b). While KMO, part of the kynurenine pathway, remained unaffected, lactate reduced HAAO, an enzyme linked to neurotoxic by-product quinolinic acid (Figures 4a,g and S3b). Additionally, lactate enhanced gut 5-HT synthesis (Figure 4h-j). Overall, lactate decreases the conversion of tryptophan to kynurenine in the colon and activates the 5-HT pathway by altering the expression of key enzymes.

(a) Volcano plot showing differentially expressed genes (DEG) between control and lactate-treated groups, highlighting upregulated (red) and downregulated (green) genes with absolute fold change >2.0 and *p* < 0.05. (b) Colon tissue sections stained with DAPI (left), IDO1 (middle), and merge images (right). Scale bar: 200 μm. (c) Fluorescence area fraction of IDO1 in colon tissue. (d) Schematic diagram illustrating the regulation pathways of tryptophan metabolism. Abbreviations: Tryptophan (TRP), Kynurenine (Kyn), 3-Hydroxy anthranilic acid (3HAA), Quinolinic acid (QA), indoleamine 2,3-dioxygenase (IDO), tryptophan 2,3-dioxygenase (TDO), kynurenine 3-monooxygenase (KMO), Kynureninase (KYNU), 3-Hydroxyanthranilate 3,4-dioxygenase (HAAO). (e) Quantitative RT-PCR analysis of *Ido1* and *Tph1* expression in mouse colon relative to 18S rRNA. (f) Pearson correlation analysis of gene expression between *Tph1* and *Ido1*. (g) Western blot analysis of enzymes involved in tryptophan metabolism. (h) Representative images of colon tissue stained for chromogranin A (CgA) (left), 5-HT (middle) and merged (right) images. Scale bar: 100 μm. (i) Quantitative assessment of 5-HT+ cells/CgA+ cells in the colon. (j) Measurement of serum 5-HT levels in mice. (k) Protein expression analysis and quantification analysis of MCT1 in mouse colon via Western blot. (l) Spearman’s rank correlation analysis between *Ido1* expression and the *Slc16a1* gene in terminal colon and small intestine samples from the healthy individuals by GEPIA. Data are presented as mean ± SD, with significance indicated by *p < 0.05; **p < 0.01; ***p < 0.001.

The Monocarboxylate transporter 1 (MCT1) of the Slc16a family is crucial for cellular lactate transport.^36–38^ Previous studies have shown that exercise promotes MCT1 upregulation, which we have also observed in lactate regulation (Figure 4k).^39^ Moreover, an inverse correlation was identified between *Slc16a1* and *Ido1* in colon and small intestine samples from healthy individuals by Gene Expression Profiling Interactive Analysis (GEPIA) (Figure 4l). This suggests that lactate may facilitate intercellular lactate transfer by upregulating MCT1, which in turn regulates tryptophan metabolism.

### Lactate and *A.* *muciniphila* co-regulate tryptophan metabolism

**Figure 5.**
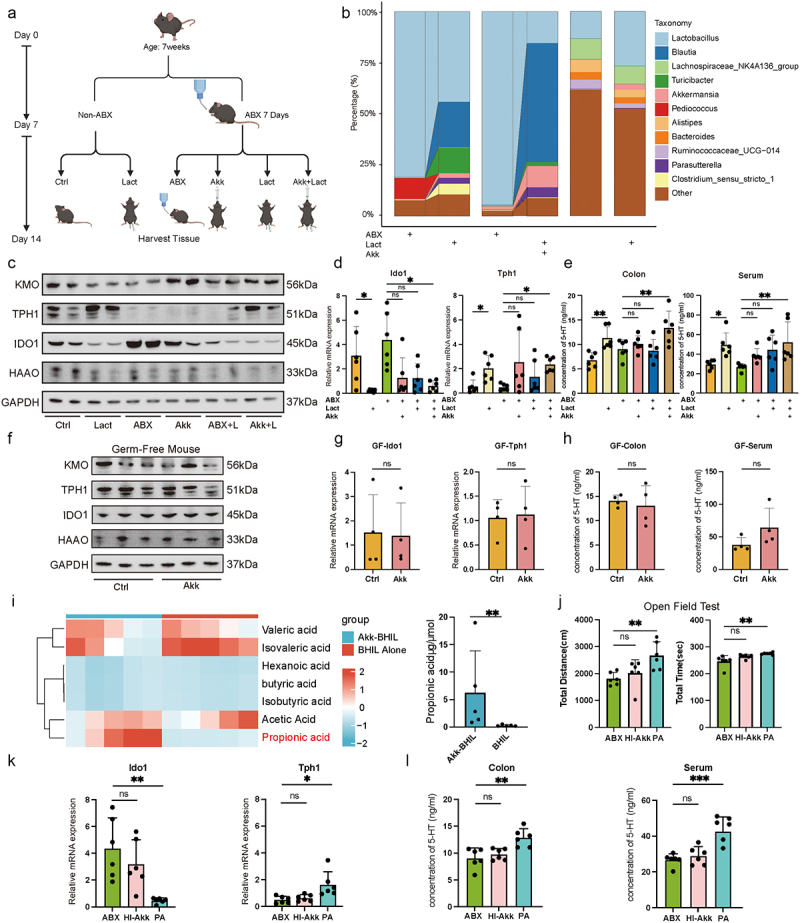
Lactate and *A. muciniphila* regulate the directional shift of tryptophan metabolism.

Given the active participation of symbiotic microbiota in the lactate cycle and their potential contribution to anxiolytic effects, we further explored the role of specific microorganisms (Figures 1c,e). We focused on *Akkermansia*, a species associated with anxiety and significantly influenced by lactate (Figures 1c, 3d). Mice underwent a seven-day antibiotic treatment, followed by the administration of *Akkermansia muciniphila (A. muciniphila)* in conjunction with lactate (Figures 5a, S3c). We found that even after antibiotic-induced microbiota depletion, lactate supplementation increased the abundance of *Akkermansia* (Figure 5b). The administration of *A. muciniphila* enhanced the alpha diversity of the gut microbiota and contributed to restoring intestinal microecology (Figures 5b, S3d). We compared the effects on 5-HT production after germ depletion followed by the supplementation of *A. muciniphila*, lactate, or combined. The results showed that the co-supplementation of *A. muciniphila* and lactate significantly affected the expression of IDO1 and TPH1 and increased 5-HT levels both locally in the colon and in the circulation (Figures 5c-e，S3e-f). To exclude the residual effects of antibiotics and the influence of microbiota changes, we performed mono-colonization with *A. muciniphila* in germ-free mice (Figure S3g). However, *A. muciniphila* alone had no significant effect on tryptophan metabolism (Figures 5f-h, S3h). In the jejunum, where the microbial colonization is relatively sparse,^40^ supplementation with lactate did not improve the activity of the 5-HT system either (Figures S4a-d). Collectively, lactate increased the abundance of *Akkermansia*, and their coexistence more effectively regulated 5-HT production.

(a) Experimental design protocol for the antibiotic reduction and gavage model in mice (*n* = 6). (b) Comparative analysis of gut microbial composition at the genus level across different groups. (c) Western blot analysis showing the expression of key enzymes involved in tryptophan metabolism in the mouse colon. (d) Quantitative RT-PCR analysis of *Ido1* and *Tph1* in mouse colon, normalized to 18S rRNA. (e) Measurement of 5-HT levels in the mouse colon and serum. (f) Western blot analysis showing the expression of key enzymes involved in tryptophan metabolism in the colon of germ-free mouse (*n* = 4). (g) Quantitative RT-PCR analysis of *Ido1* and *Tph1* in germ-free mouse colon, normalized to 18S rRNA. (h) Measurement of 5-HT levels in the germ-free mouse colon and serum. (i) Concentration of SCFAs in the supernatant of BHI medium supplemented with 60% sodium lactate, with or without *A. muciniphila*. (j) Total movement distance and activity time of mice in open field. (k) Quantitative RT-PCR analysis of *Ido1* and *Tph1* in mouse colon, normalized to 18S rRNA. Heat-inactivation *A. muciniphila* : HI-Akk, Propionic acid: PA. (l) Measurement of 5-HT levels in the mouse colon and serum. Data are presented as mean ± SD, with significance indicated by *p < 0.05; **p < 0.01; ***p < 0.001; ns, no significance.

Next, we further investigated how *A. muciniphila* participates in the regulation of tryptophan metabolism. Studies have shown that *Akkermansia* can effectively metabolize lactate as a carbon source,^27^ and is an important source of SCFAs in the gut.^41^ Therefore, we performed targeted SCFA metabolite analysis on the supernatant of *A. muciniphila* cultures supplemented with lactate. Addition of lactate significantly increased the concentration of propionate in the supernatant (Figure 5i). To identify the active component of *A. muciniphila*, we also compared the effects of heat-inactivated *A. muciniphila* and propionate on mouse metabolism. Propionate enhanced the activity of the 5-HT system in mice and increased their movement in the center area of the open field (Figure 5j-l). Supplementation with heat-inactivated *A. muciniphila* did not result in significant improvement, confirming that propionate may be an effective mechanism by which *A. muciniphila* reduces anxiety. Taken together, these results suggested that the propionate, produced by *A.* *muciniphila* in a lactate environment, as the key mediator for anti-anxiety effect. Propionate might be the effective anti-anxiety component produced by *A. muciniphila* metabolism.

### Lactate maintained intestinal barrier integrity dependent on gut microbiota

**Figure 6.**
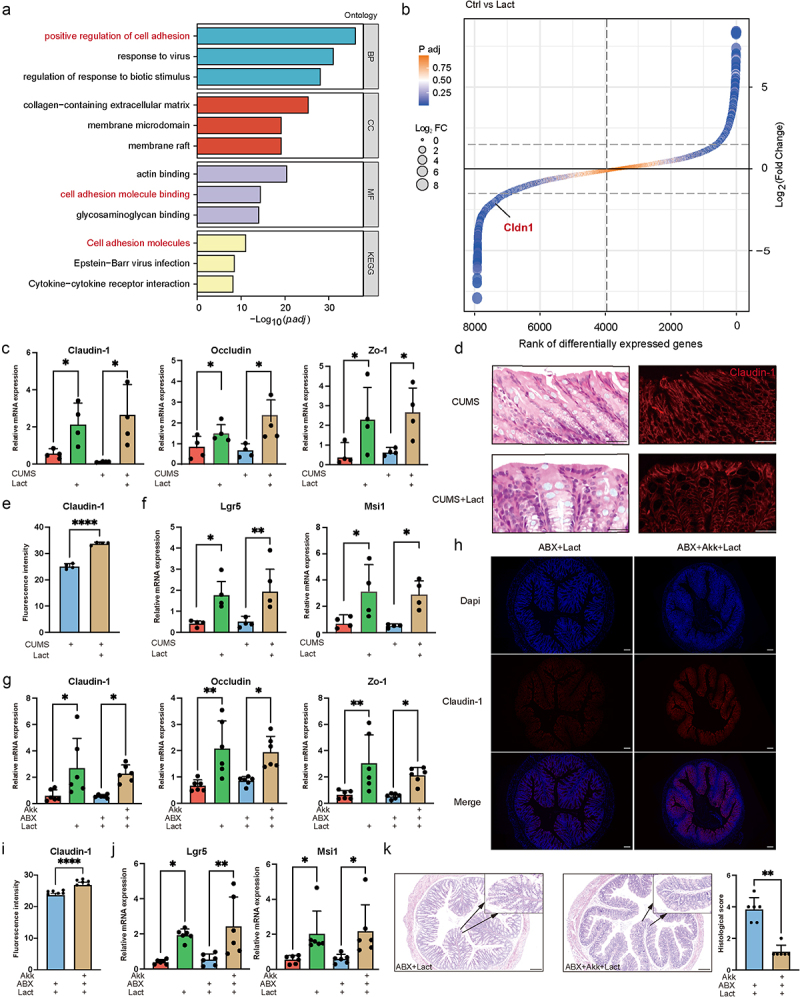
Lactate relies on gut microbiota to maintain intestinal barrier integrity.

To delve deeper into how lactate contributes to intestinal homeostasis, we conducted Gene Ontology (GO) and the Kyoto Encyclopedia of Genes and Genomes (KEGG) enrichment analysis on mouse colon. Differential gene analysis revealed that lactate affected the cell adhesion process (Figure 6a). Notably, the gene *Cldn1*, which encodes Claudin-1, exhibited a significant increase alongside markers of intestinal permeability, *Occludin*, and *Zo-1* (Figures 6b-e, S4e). *Lgr5* and *Msi1* levels, associated with intestinal stem cell regeneration, were also significantly elevated by lactate, which is vital for maintaining the integrity of the gut barrier (Figure 6f).^42^ We further evaluated the role of microbes in restoring the gut barrier. The synergistic use of lactate and *A. muciniphila* effectively restored the intestinal barrier (Figures 6g-k). A robust gut barrier is a fundamental determinant of mental health.^43,44^ These results suggest that the collaboration between lactate and *A. muciniphila* is pivotal in maintaining gut barrier integrity, preventing ecological imbalances, and benefiting the host’s mental health.

(a) Gene Ontology (GO) and Kyoto Encyclopedia of Genes and Genomes (KEGG) functional enrichment analyses of differentially expressed genes between control and lactate-treated mouse colon. (b) Ranking of differentially expressed genes (DEGs) in the colon, with a focus on Cldn1. (c) qPCR analysis of *Claudin-1*, *Occludin*, and *Zo-1* mRNA expression in mouse colonic tissue, normalized to 18S rRNA. (d-e) Hematoxylin and eosin (H&E) staining and immunofluorescence staining for Claudin-1 in mouse colonic tissue and its statistical graph (e) Scale bar: 50 μm. (f) mRNA expression of intestinal stem cell markers, including *Lgr5* and *Msi1* normalized to 18S rRNA. (g) mRNA expression of *Claudin-1*, *Occludin*, and *Zo-1* in the intestinal tract of mice following *A. muciniphila* administration, normalized to 18S rRNA. (h-i) Representative images of colon tissue stained for DAPI (top), Claudin-1 (middle) and merged (bottom) images and its statistical graph (i) Scale bar: 200 μm. (j) mRNA expression profiles of intestinal stem cell markers of mice following *A. muciniphila* administration, normalized to 18S rRNA. (k). Hematoxylin and eosin (H&E) staining of mouse colonic tissue and statistical graph. Scale bar: 200 μm. Data are presented as mean ± SD, with significance indicated by *p < 0.05; **p < 0.01; ***p < 0.001.

## Discussion

**Figure 7.**
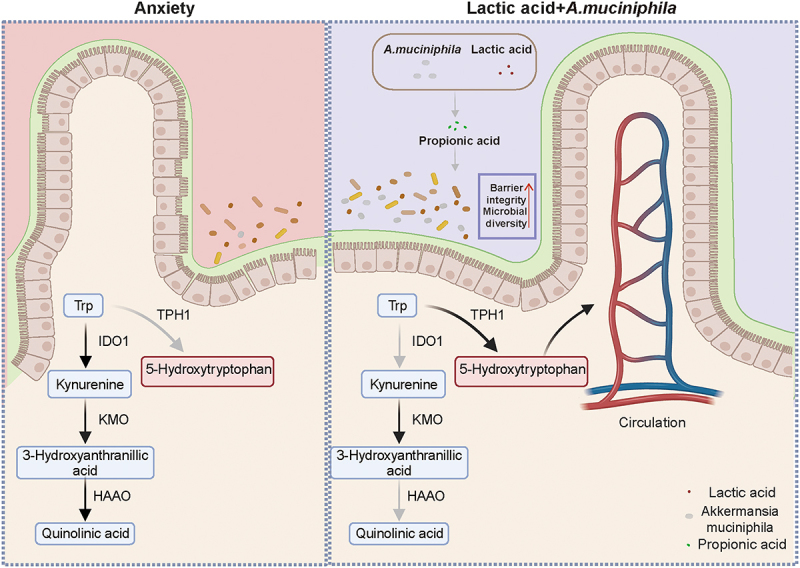
Schematic illustration of the regulatory role of lactate and *A. muciniphila* on tryptophan metabolism.

Given the controlled conditions of Chinese athletes in our study, we focused on how the gut microbiota influences anxiety. The results suggest a negative correlation between anxiety levels and the abundance of *Akkermansia*. We found that lactate serves as a key molecule linking microbiota to the anxiety regulation. In a lactate-enriched environment, the abundance of *A. muciniphila* significantly increased, accompanied by an elevated concentration of propionate. This process markedly altered the expression of IDO1 and TPH1, shifting tryptophan metabolism from the kynurenine pathway (KP) to 5-HT production. The synergistic interaction between lactate and microbiota enhances intestinal barrier integrity (Figure 7).

Peripherally circulating 5-HT is derived from tryptophan metabolized in the gut. Tryptophan is an essential amino acid obtained exclusively through diet, with its metabolites serving critical functions as neurotransmitters and signaling molecules.^45^ Activation of IDO1 leads to the accumulation of Kyn and its derivatives, including the neurotoxic metabolite QA.^46^ Elevated levels of QA and an increased Kyn/TRP ratio are commonly observed in psychiatric disorders.^47^ Our study demonstrated that anxiety in mice increased IDO1 and HAAO expression, while *Tph1* was inversely correlated with *Ido1* levels. These results indicate that tryptophan metabolism is susceptible to modulation, potentially disturbing neurotransmitter function and contributing to mental health disorders.^33^ Recent studies have highlighted tryptophan metabolism as a therapeutic target for mental health. Supplementation with tryptophan or colonization by specific microorganisms, such as *Lactococcus lactis* or *Lactobacillus rhamnosus GG*, has been shown to effectively restore metabolic balance and improve phenotypic outcomes.^48–50^ Additionally, we found that the combination of lactate and *A. muciniphila* effectively regulated the 5-HT system. This indicates that microorganisms and their co-metabolites can collaborate in regulating the host’s metabolic balance. Further research should explore whether similar synergistic interactions have additional effects on the host’s physiological state.

Our findings indicate that the metabolic regulation of tryptophan by lactate is intricately dependent on normal microbial activity, with *A. muciniphila* playing a particularly significant role. The presence of gut microbiota is crucial for the enhanced functionality of the gut serotonin system, as evidenced by the limited effects observed in the jejunum, where microbial colonization is sparse. Fluctuations in gut-derived 5-HT can be modulated by microorganisms, potentially through their by-products such as short-chain fatty acids (SCFAs) or soluble factors produced by spore-forming bacteria, which act as direct signals influencing the secretory capacity of enterochromaffin cells.^8,51^ Additionally, the outer membrane protein Amuc_1100 from *A. muciniphila* regulates the host intestinal 5-HT system via Toll-like receptor 2 (TLR2).^52,53^ These findings support the hypothesis that the 5-HT pathway is directly modulated by microbial metabolites. However, it remains unclear whether the upstream components of 5-HT synthesis are similarly affected. Previous studies have shown that IDO1 activation in the colon has a greater impact on tryptophan homeostasis compared to the small intestine, likely due to the presence of resident microbiota.^33^ Our research confirmed that the supplementation of lactate in the culture medium significantly increased propionate levels, likely because lactate provided a carbon source for *A. muciniphila*. Propionate may be an important component by which *A. muciniphila* regulates anxiety, as heat-inactivated *A. muciniphila* exhibited no effect. Studies have shown that the propionate-producing capacity of *A. muciniphila* is associated with intestinal epithelial development.^54^ Our results revealed that lactate provides a beneficial proliferative environment for *A. muciniphila*. Meanwhile, *A. muciniphila* is sufficient to contribute to lactate in improving host metabolic imbalances .

Athletes generate higher levels of lactate, which can be metabolized in the gut, creating a special environment. *Veillonella* and *Akkermansia*, key microbes involved in lactate circulation, were found in higher abundance in athletes.^21,55^ Interestingly, these two microbes were particularly abundant in team members exhibiting lower anxiety within our athlete cohort. We observed a higher abundance of genes associated with lactate metabolism in non-anxious athletes, suggesting that microbial lactate cycling capacity may play a role in modulating anxiety. Additionally, microbes like *Veillonella*, which recycles lactate, enhance exercise performance by converting lactate to propionic acid.^21^ Our findings indicate that lactate supplementation alone improved exercise performance in mice. This is likely because lactate provides metabolic substrates for *A. muciniphila*, promoting propionate production, which in turn enhances endurance and balance in mice. Further research into the interactions between lactate and the gut microbiome could offer valuable insights into the mental and physical health of elite athletes, contributing to new perspectives in sports psychology.

## Limitations of the study

The present study has some limitations. Firstly, the beneficial effects of lactate and *A. muciniphila* need to be validated in larger human cohorts. Second, further research is required to explore how metabolites and microbes interact in the regulation of tryptophan metabolic pathways involved in anxiety, including a detailed analysis of the active components of *A. muciniphila*.

## Conclusions

In conclusion, our *in vivo* data supported that enteric lactate and *A. muciniphila* collaboratively facilitates tryptophan metabolism to 5-hydroxytryptamine. These findings provide new insights into the complex interplay between gut metabolites and mental health, offering promising avenues for microbiota-targeted therapeutic interventions in anxiety management. Our findingsexpand the understanding of lactate by demonstrating its direct involvement in anxiety regulation and its ability to create a more favorable microbial environment.

## Supplementary Material

Supplemental Material

## Data Availability

The datasets generated and analyzed during the current study are available in the China National Center for Bioinformation portal repository (subPRO019926, https://ngdc.cncb.ac.cn/sso/login).
