# Supplementary material for: Gut-derived lactic acid enhances tryptophan to 5-hydroxytryptamine in regulation of anxiety via Akkermansia muciniphila
Source: Gut Microbes. 2025 Jan 9;17(1):2447834. doi: 10.1080/19490976.2024.2447834 (PMC11730363; doi:10.1080/19490976.2024.2447834)
Supplement: Supplemental Material [file KGMI_A_2447834_SM5757.docx]

**SUPPLEMENTAL INFORMATION**

Supplemental information includes 4 figures and 2 tables.

**SUPPLEMENTAL FIGURE**

**
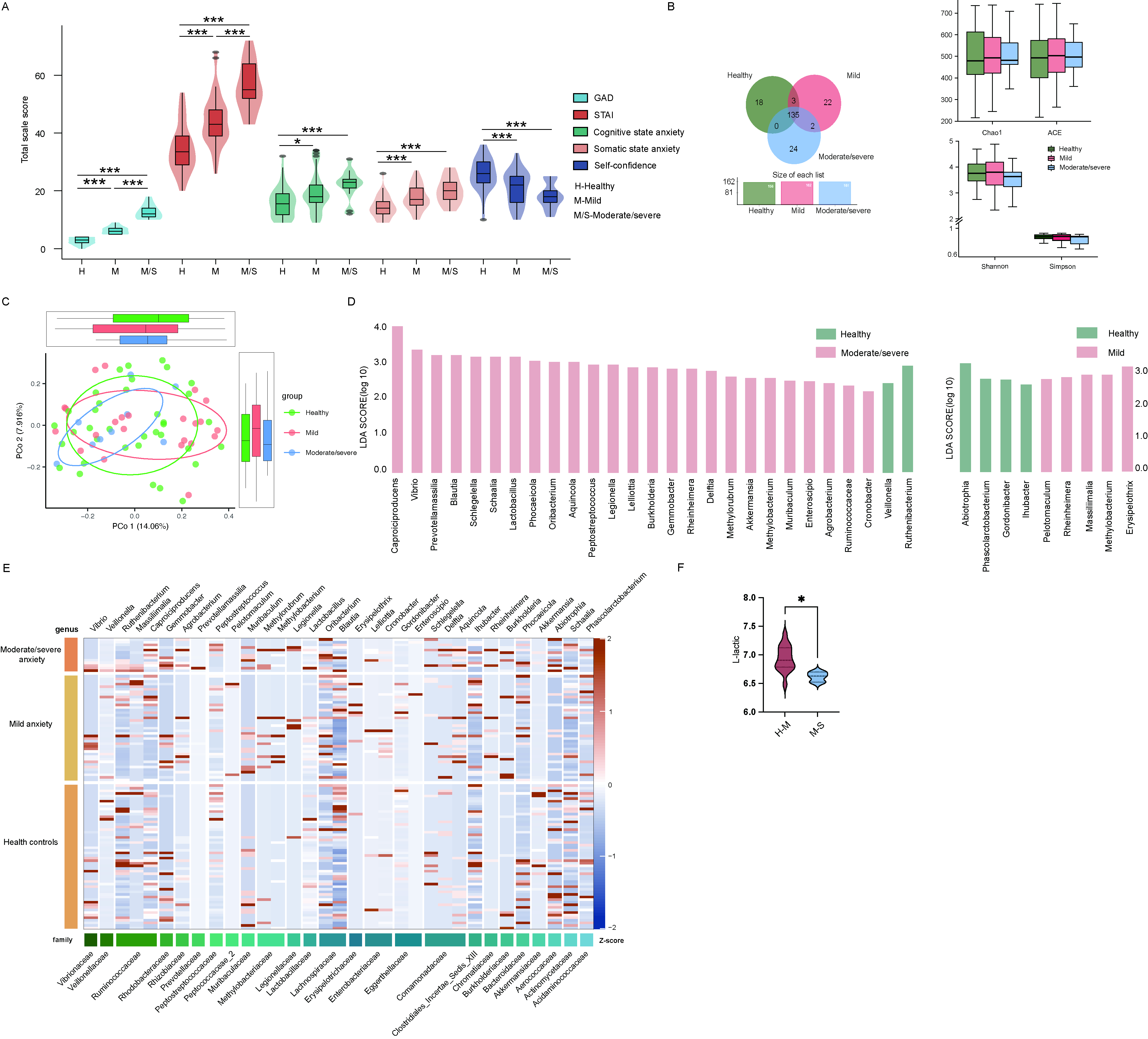
**

Figure. S1 Anxiety in athletes linked to gut microbiome and lactic acid metabolism

A. Psychometric scale assessment of healthy, mildly anxious, and moderate/severe anxious shooters: GAD (Generalized Anxiety Disorder Scale); STAI (State-Anxiety Inventory trait Version); CSAI (Competitive State Anxiety Inventory, including Cognitive state anxiety, Somatic state anxiety and Self-confidence). H: healthy; M: mild; M/S: moderate/severe.

B. Venn diagram showing shared amplicon sequence variants (ASVs) across healthy, mild, and moderate/severe anxiety groups. Alpha-diversity indices (Chao1, ACE, Shannon, Simpson) represented as bar charts.

C. Principal coordinate analysis (PCoA) of gut microbiota Beta-diversity in athlete based on Bray-Curtis dissimilarity.

D. LEfSe LDA histograms showing taxa with significant differences in abundance between groups (LDA score > 2.0).

E. Heatmap illustrating the distribution of distinct microbial families and genera that differ among the groups (LDA > 2.0).

F. Fecal lactate content in both groups: H-M (Healthy-Mildly anxiety group) and M-S (Moderate-Severe anxiety group). Significance levels are indicated as follows: * *p*<0.05, ** *p*<0.01, *** *p*<0.001.


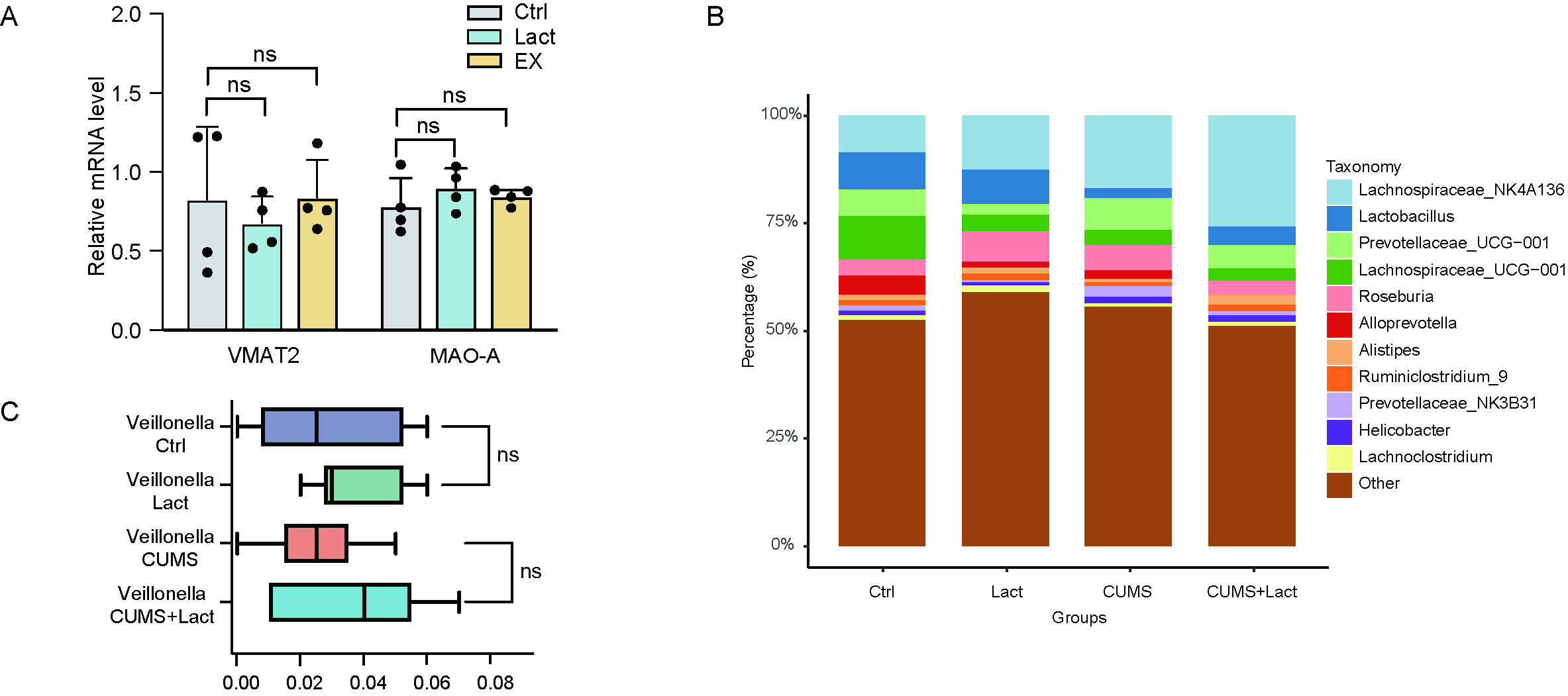


Fig. S2 Comparative microbial composition profiles in murine models

A. Gene expression profiles in the mouse prefrontal cortex for serotonin metabolism pathways, normalized to 18S rRNA, including vesicular monoamine transporter 2 (VMAT2) and monoamine oxidase A (MAO-A).

B. Relative abundance of microbial taxa at the genus level for each group.

C. Relative abundance measurements for *Veillonella* in mice, ns, no significance.


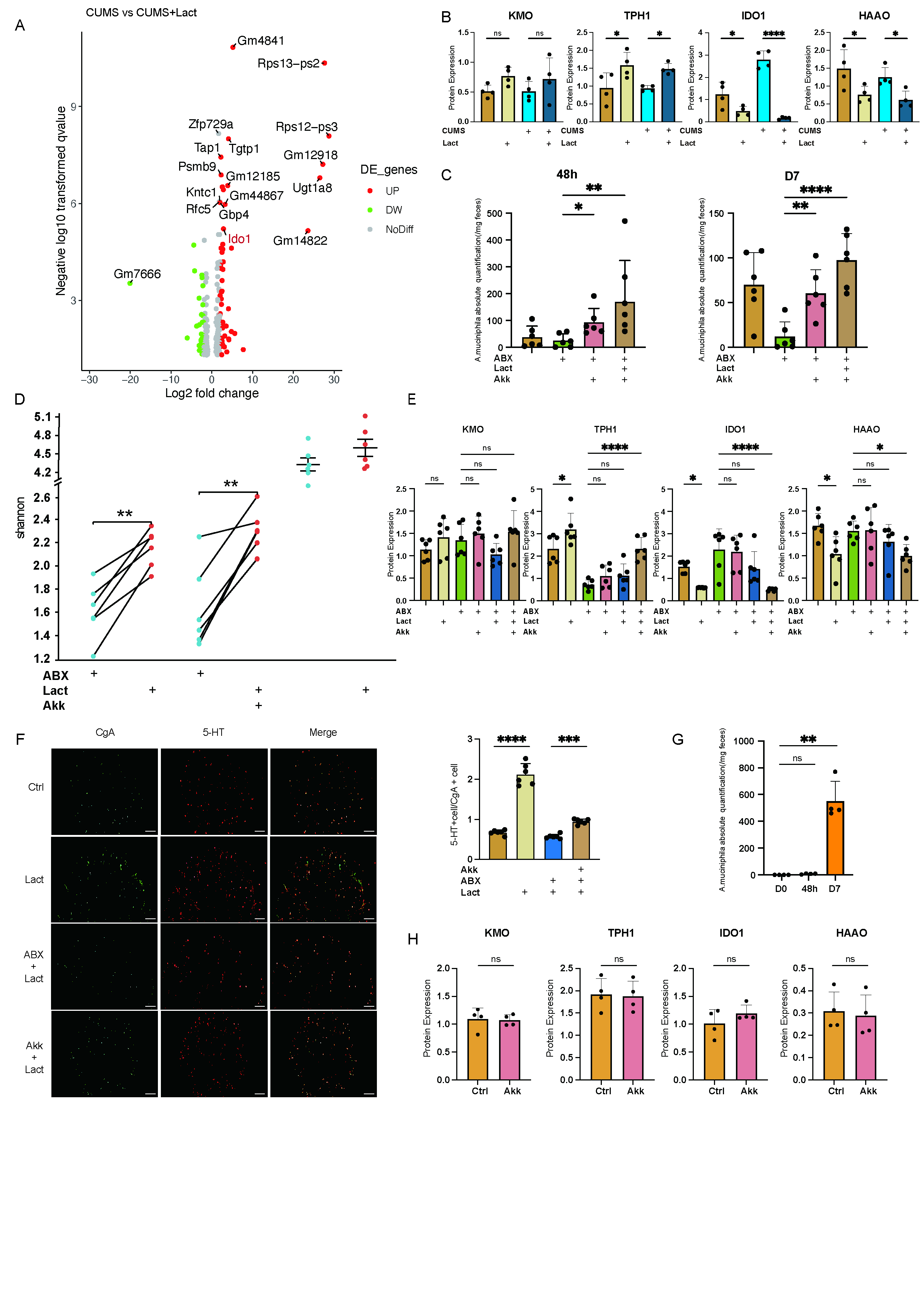


Fig. S3 Transcriptome analysis of the mouse colon and *A.* muciniphila colonization

A. Volcano plot showing differentially expressed genes (DEGs) between the CUMS and CUMS-Lact groups. Upregulated genes are marked in red, downregulated genes in green, with a fold change threshold >2.0 and *p* < 0.05.

B. Protein quantification results of the key enzymes involved in tryptophan metabolism in CUMS mouse colon.

C. Precise quantification of *A.* muciniphila in stool of mice following intragastric administration of *A.* muciniphila (48h and 7 days).

D. Alpha-diversity indices (Shannon) represented as bar charts.

E. Protein quantification results of the key enzymes involved in tryptophan metabolism in *A.* muciniphila gavage mouse colon.

F. Representative images of colon tissue stained for CgA (left), 5-HT (middle) and merge (right) image. Scale bar: 200μm. Quantitative assessment of 5-HT+ cells/CgA+ cells of the colon.

G. Precise quantification of *A.* muciniphila in stool of germ-free mice following intragastric administration of *A.* muciniphila (48h and 7 days).

H. Protein quantification results of the key enzymes involved in tryptophan metabolism in grem-free mouse colon. Data are presented as mean ± SD, with significance indicated by *, *p*<0.05; **, *p*<0.01; ***, *p*<0.001; ns, no significance.


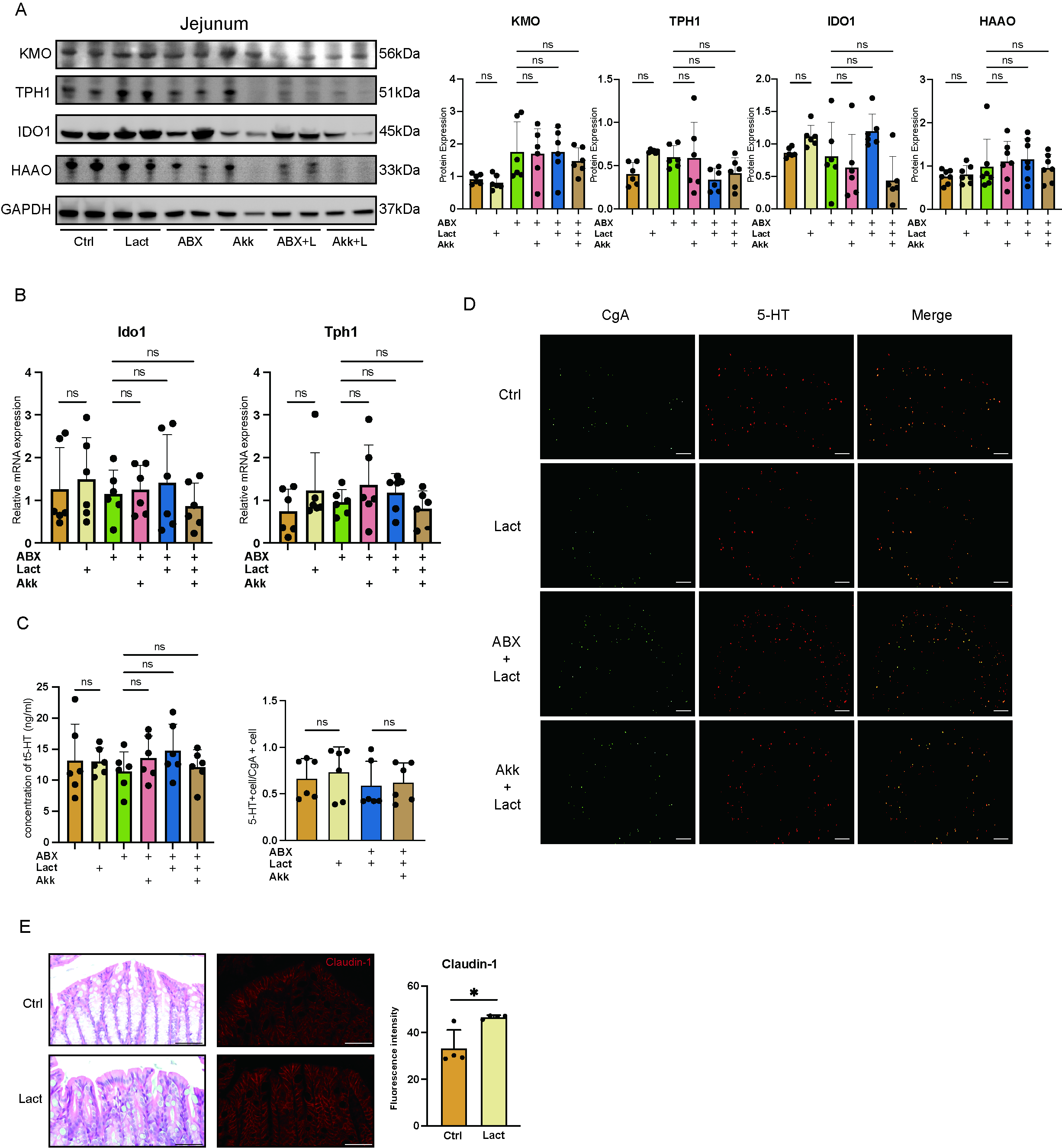


Fig. S4 Gene expression of 5-HT system in mouse jejunum

A. Western blot analysis of key enzymes involved in tryptophan metabolism and their quantification results in the mouse jejunum.

B. Quantitative RT-PCR of *Ido1* and *Tph1* in mouse jejunum, normalized to 18S rRNA.

C. Measurement of 5-HT levels in the mouse jejunum.

D. Representative images of jejunum tissue stained for chromogranin A (CgA) (left), 5-HT (middle) and merged images (right) and their quantitative assessment results. Scale bar: 200μm.

E. Hematoxylin and eosin (H&E) staining and immunofluorescence staining for Claudin-1 in mouse colonic tissue and its statistical graph. Scale bar: 50μm. Data are presented as mean ± SD. Significance levels are indicated as follows: *, *p*<0.05; **,, *p*<0.01; *** *p*<0.001; ns, no significance.

**SUPPLEMENTARY TABLE 1**

|  | | Healthy  (n=56) | Mild  (n=41) | Moderate/severe  (n=13) | Statistics |
| --- | --- | --- | --- | --- | --- |
|  |  | Mean±SD | Mean±SD | Mean±SD |  |
| **Age(year)** | | 19.18±4.13 | 20.63±3.43 | 20.46±3.82 | *p* = 0.0630 |
| **Gender** | Male | 33(58.93%) | 29(70.73%) | 4(30.77%) | *p* = 0.036 |
|  | Female | 23(41.07%) | 12(29.27%) | 9(69.23%) |  |
| **BMI (kg/m^2^)** | | 23.20±3.72 | 23.59±3.63 | 23.64±3.16 | *p* = 0.8475 |
| **SYS (mmHg)** | | 105.82±15.92 | 109.76±14.14 | 109.69±7.95 | *p* = 0.2749 |
| **DBP (mmHg)** | | 65.79±11.04 | 67.76±10.25 | 65.85±9.35 | *p* = 0.6440 |
| **WHR** | | 0.83±0.06 | 0.83±0.05 | 0.82±0.04 | *p* = 0.7288 |
| **Diet** | Calories (kcal/d) | 1975.66±580.86 | 2129.83±592.66 | 1820.91±579.38 | *p* = 0.2005 |
|  | Carbohydrates (g/d) | 256.13±93.20 | 273.49±103.71 | 250.66±70.74 | *p* = 0.8502 |
|  | Protein (g/d) | 100.32±38.94 | 107.69±46.63 | 78.86±40.88 | *p* = 0.0770 |
|  | Fat (g/d) | 61.70±28.92 | 67.87±24.84 | 56.58±31.57 | *p* = 0.1858 |
|  | Dietary fiber (g/d) | 7.86±4.87 | 8.62±4.47 | 12.34±5.34 | *p* = 0.0188 |

Table S1 Participant Characteristics in Athlete

Abbreviations: BMI=Body mass index; SYS=Systolic blood pressure; DBP=Diastolic blood pressure; WHR=Waist-to-hip ratio; SD=Standard Deviation.

**SUPPLEMENTARY TABLE 2**

Primer sets for qPCR analysis.

| **Primer** | **Primer sequence** |
| --- | --- |
| ***Ido1-F*** | ATCCTTGAAGACCACCAC |
| ***Ido1-R*** | AGGCAGATTTCTAGCCAC |
| \| ***VMAT2-F*** \| \| --- \| | TGCTGAAGGACCCATACATTC |
| ***VMAT2-R*** | CACATGGTCTCCATCATCCA |
| ***Claudin1-F*** | TGCCCCAGTGGAAGATTTACT |
| ***Claudin1-R*** | CTTTGCGAAACGCAGGACAT |
| ***Occludin-F*** | TTCAGGTGAATGGGTCACCG |
| ***Occludin-R*** | AGATAAGCGAACCTGCCGAG |
| ***Zo-1-F*** | ACCTCTGCAGCAATAAAGCAG |
| ***Zo-1-R*** | GAAATCGTGCTGATGTGCCA |
| ***Lgr5-F*** | GGACCAGATGCGATACCGC |
| ***Lgr5-R*** | CAGAGGCGATGTAGGAGACTG |
| ***Msi1-F*** | TAAAGTGCTGGCGCAATCG |
| ***Msi1-R*** | TCTTCGTCCGAGTGACCATCT |
| ***Tph2-F*** | GTGACCCTGAATCCGCCTG |
| ***Tph2-R*** | GGTGCCGTACATGAGGACT |
| ***MAOA-F*** | TCTGTTGGACAAAAACTGCTC |
| ***MAOA-R*** | ATTTGGCCAGAGCCACCTA |
| ***Tph1-F*** | GAAGACAACATCCCGCAACT |
| ***Tph1-R*** | AAAGGCTAACCCCGACAGA |
| **18S rRNA-F** | CGGCTACCACATCCAAGGAA |
| **18S rRNA-R** | GCTGGAATTACCGCGGCT |
